# Supplementary material for: Theoretical Investigation of the Coronavirus SARS-CoV-2 (COVID-19) Infection Mechanism and Selectivity
Source: Molecules. 2022 Mar 24;27(7):2080. doi: 10.3390/molecules27072080 (PMC9000624; doi:10.3390/molecules27072080)
Supplement: Supplementary file 1 [file molecules-27-02080-s001.zip › molecules-1609662-supplementary-final.pdf]

SUPPLEMENTARY DATA

A)

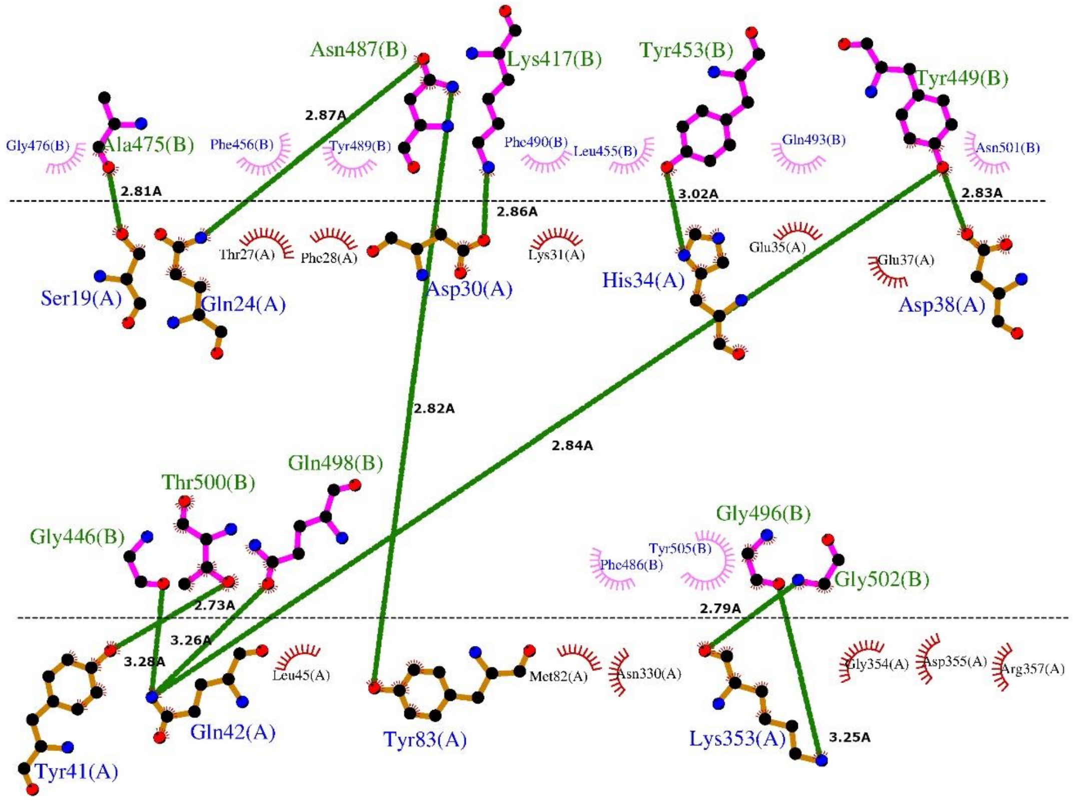

B)

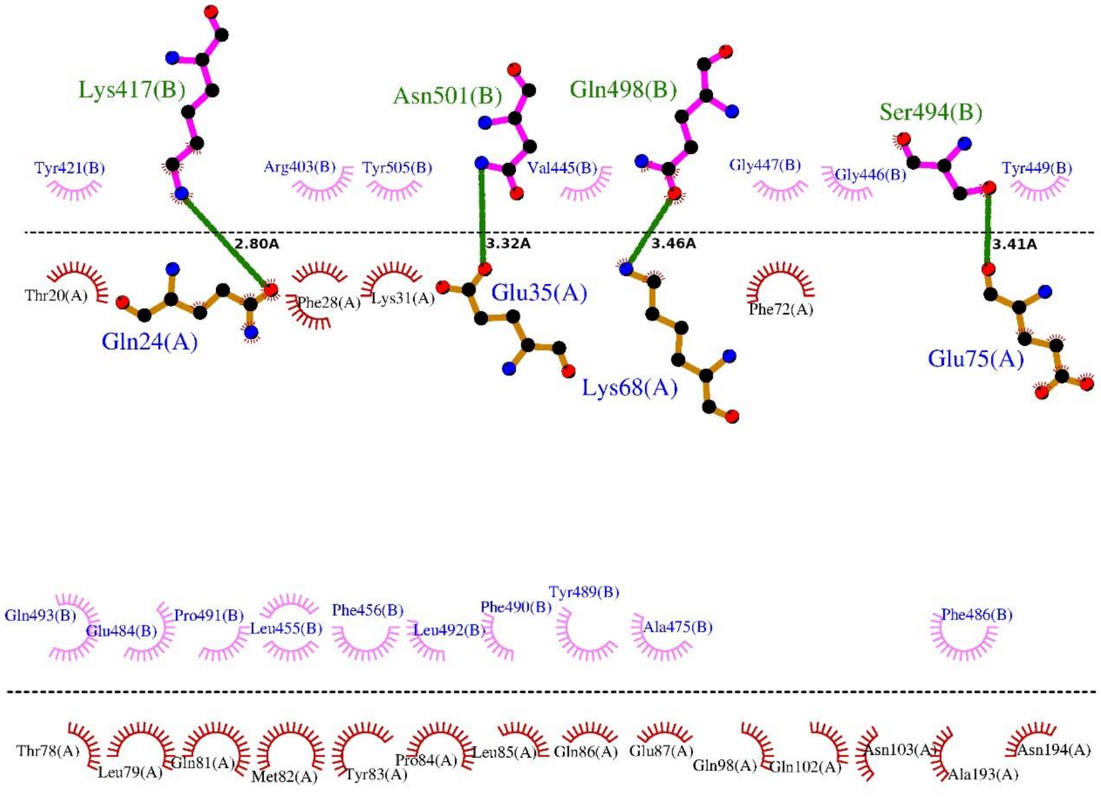

Figure 1. 2D plot of the SarsCov2 spike receptor – ACE2 interface. A) 6LCY PDB structure. B) UNRES MD model [62].

[62] Laskowski R A, Swindells M B (2011). LigPlot+: multiple ligand-protein interaction diagrams for drug discovery. *J. Chem. Inf. Model.*, **51**, 2778-2786.

UNRES INPUT OPTIONS

“”

```
Multi MREMD
SEED=-3059743 MD rescale_mode=2           PDBSTART timlim=2800.0             &
pdbref refstr BOXX=200 BOXY=200 BOXZ=200 tormode=2 cart
nstep=8000000  dt=0.1 ntwe=10000 ntwx=10000    lang=1             t_bath=300 &
damax=20.0 EDRIFTMAX=20.0 reset_moment=10000 reset_vel=0  cart
WSC=0.81230E+00 WSCP=0.12042E+01 WELEC=0.87690E+00 WBOND=0.10000E+01           &
WANG=0.19773E+01 WSCLOC=0.63657E-01 WTOR=0.14154E+01 WTORD=0.00000E+00         &
WCORRH=0.00000E+00 WCORR5=0.00000E+00 WCORR6=0.00000E+00 WEL_LOC=0.36638E-01   &
WTURN3=0.14511E+01 WTURN4=0.00000E+00 WTURN6=0.00000E+00 WSCCOR=0.00000E+00    &
WVDWPP=0.33393E+00 WHPB=0.10000E+01 WSCP14=0.00000E+00                         &
CUTOFF=7.00000 WCORR4=0.00000 LIPSCALE=1.0 WLT=1.0
./model.pdb
0
0
```
